# Virtual Multiplexing Chamber-Based Digital PCR for Camel Milk Authentication Applications

**DOI:** 10.3390/mi14081619

**Published:** 2023-08-17

**Authors:** Jinchao Li, Jingmeng Cheng, Shanshan Li, Jie Jayne Wu, Junwei Li

**Affiliations:** 1 Hebei Key Laboratory of Smart Sensing and Human-Robot Interactions, School of Mechanical Engineering, Hebei University of Technology, Tianjin 300130, China; 2 State Key Laboratory of Reliability and Intelligence of Electrical Equipment, Hebei University of Technology, Tianjin 300130, China; 3 Department of Electrical Engineering and Computer Science, The University of Tennessee, Knoxville, TN 37996, USA; 4 Institute of Biophysics, School of Health Sciences and Biomedical Engineering, Hebei University of Technology, Tianjin 300401, China

**Keywords:** microfluidics, digital polymerase chain reaction, camel authentication

## Abstract

In this work, we proposed a chamber-based digital PCR (cdPCR) microfluidic device that is compatible with fluorescence imaging systems for milk adulteration detection. The device enables the digitalization of PCR reagents, which are loaded into microchambers, and subsequent thermocycling for DNA amplification. Then, fluorescence images of the microchambers are captured and analyzed to obtain the total number of positive chambers, which is used to calculate the copy numbers of the target DNA, enabling accurate quantitative detections to determine intentional milk adulteration from accidental contaminations. The validation of this device is performed by camel milk authentication. We performed 25,600-chamber virtual multiplexing cdPCR tests using 40 × 40 chamber devices for the detection of DNA templates extracted from pure or mixed milk with different dilutions. Then, the cdPCR chip was used to authenticate blind milk samples, demonstrating its efficacy in real biotechnical applications.

## 1. Introduction

Camel milk is a highly valued commodity due to its unique nutritional properties and potential health benefits. However, the increasing demand for camel milk has led to many instances of adulteration with other milk types, such as cow or buffalo milk, to increase profits [1,2]. Therefore, there is a demand to develop an approach or device that provides ready access for camel milk authentication. Among the state-of-the-art methods, polymerase chain reaction (PCR) technology, which can multiply the target deoxyribonucleic acid (DNA) exponentially, offers significant advantages of strong specificity, high sensitivity, and good repeatability [3]. Various PCR-based methodologies, including qualitative PCR [4,5,6], quantitative PCR [7,8,9,10,11], and competitive real-time PCR [12,13], have been developed and are widely used in many fields. However, traditional PCR suffers from limitations in absolute quantitative detection. To overcome these limitations, digital PCR has emerged [14,15], which is an absolute quantitative nucleic acid amplification technology that directly obtains the copy number of DNA molecules without using calibration standards. So this process is faster, more accurate, and repeatable [16,17]. Moreover, digital PCR can detect DNA samples with very low copy numbers, greatly improving detection sensitivity.

The advancement of microfluidic technology has overcome the fabrication bottleneck of digital PCR, leading to the accelerated development of digital PCR devices and methods. [18]. Currently, microfluidics-based digital PCR mostly falls into two main groups: chamber-based digital PCR (cdPCR) [19,20,21] and droplet-based digital PCR (ddPCR) [22,23,24]. Commercial ddPCR systems usually consist of a droplet generator, a thermocycler, and a droplet reader. The multiple instruments take up valuable lap space and require trained personnel for droplet generation. Compared with ddPCR, cdPCR has the distinct advantage of more accurate data. In addition, it also avoids stability problems such as mutual fusion and cross-contamination [25]. So cdPCR-based microfluidic devices have played a significant role in detecting milk adulteration. Nonetheless, digital PCR, including both cdPCR and ddPCR, suffers from time-consuming, labor-intensive processes, bulky instruments, as well as high costs for food adulteration applications [26]. Therefore, there is an urgent need for the development of a straightforward and cost-effective approach to milk adulteration detection.

In real applications, the economic benefits from milk adulteration would be limited once the mass ratio of cow or buffalo milk to camel milk is less than 1:9; thus, it is required to perform a quantitative analysis for unknown milk samples. The sensitivity or accuracy of cdPCR is strongly related to the chamber volume, and especially the chamber counts. The accuracy of cdPCR could be improved by increasing the number of microchambers. The straightforward approach to improve its quantitative ability is to fabricate a cdPCR with more than 20,000 chambers. However, with the increasing amount of microchambers, the FPY (first pass yield) decreases significantly. Moreover, the processes of predegassing and sample loading operation become very challenging due to increasing flow resistance. To address these problems, we proposed the ‘virtual multiplexing’ concept in this paper to obtain a 25,600 level throughput (more independent chambers stand for higher accuracy) by using 40 × 40 chamber devices.

In this study, our objective is to develop a cdPCR microfluidic device for milk authentication, which is compatible with fluorescence image read-out systems and demonstrates full functionalities of self-digitalization, reagent loading, DNA amplification, fluorescence imaging, and optical analysis. Firstly, we performed cdPCR tests to use camel–cow mixtures with different dilutions as samples. Using this device, the digitalized PCR reagents are introduced into microchambers by negative pressures with a free outlet. After a DNA amplification process, fluorescence images of microchamber arrays are read out and analyzed to obtain the number of total positive chambers. At last, the copy numbers of the target DNA can be calculated for quantitative analysis. We envision that our dPCR microfluidic chip, combined with fluorescence image read-out systems, can become an integral tool in life science and biomedicine. Compared with traditional methods for milk authentication, the cdPCR proposed in this work uses PDMS to create microchambers instead of droplets and does not require external pumps to generate droplets. It also avoids the risk of droplet fusion or breakage and cross-contaminations.

## 2. Materials and Methods

### 2.1. Preparations of Reagent, Primers and Probes

Cow milk and camel milk samples were purchased from a local market. Their DNA was extracted and purified. Seven kinds of cow/camel milk mixture samples were prepared as 0/10, 1/9, 2.5/7.5, 5/5, 7.5/2.5, 9/1, and 10/0 (unit: mL) by *v*/*v*. Washing buffer was prepared with Tris–HCl (pH 7.4–7.6, 15 mM), NaCl (25 mM), MgCl_2_ (5 mM), Na_2_HPO_4_ (15 mM), EDTA (2.5 mM) and 1% sucrose solution. Lysis buffer (pH = 8.8) was prepared with 6% SDS, 3 mmol L^−1^ MgCl_2_, 15 mmol L^−1^ Tris–HCl, 0.5% dimethyl sulfoxide, and 6% acetone. Protein precipitation buffer (pH = 5.0) was prepared with 2.35 M NH_4_Cl, 1.15 M NaCl, and 38% ethanol. All the materials are from Sigma-Aldrich (Shanghai, China).

Each group of milk samples was tested three times. The DNA was extracted by sodium dodecyl sulfate method. Firstly, each milk sample was centrifuged to remove any potential cream or supernatant, and then the centrifugal precipitates were collected from the bottom of 10 mL centrifuge tubes. Next, the precipitate was washed twice by washing buffer. Then 2 mL lysis buffer and 450 µL protein precipitation buffer were added. After a centrifugal operation, the supernatant was transferred into a brand-new tube, and 1 mL of isopropanol was added. Then, a nucleospin column was used to absorb the nucleospin silica-gel membrane. After the final elution and drying, the extracted DNA was dissolved in 80 µL TE buffer.

#### Design of Specific Primers and Probes

The specific primer and probes were designed using Primer 5.0 tool, and all the primers and probes were synthesized by Beijing Prime Tech Biotechnology Co. (Beijing, China), as shown in Table 1.

After the PCR reagents were introduced into the microchambers, PCR amplification was conducted as follows. We experimented with initial denaturation and enzyme activation (95 °C, 5 min), then we performed 30 thermal cycles, including three steps. The first step was denaturation (94 °C, 40 s); in this process, DNA was dissociated from double-stranded DNA to single-stranded DNA. The second step was annealing (55 °C, 20 s) which involves binding the primers with the complementary sequence of the single-stranded DNA template. The third step was extension (72 °C, 30 s); the purpose of this step was to synthesize a new double-stranded DNA. The heating/cooling process was carried out on the cdPCR microfluidic chip using a self-developed TEC (thermoelectric coolers) controller using an STM32 microcontroller.

### 2.2. Design and Fabrication of cdPCR Microfluidic Chip

**Figure 1:**
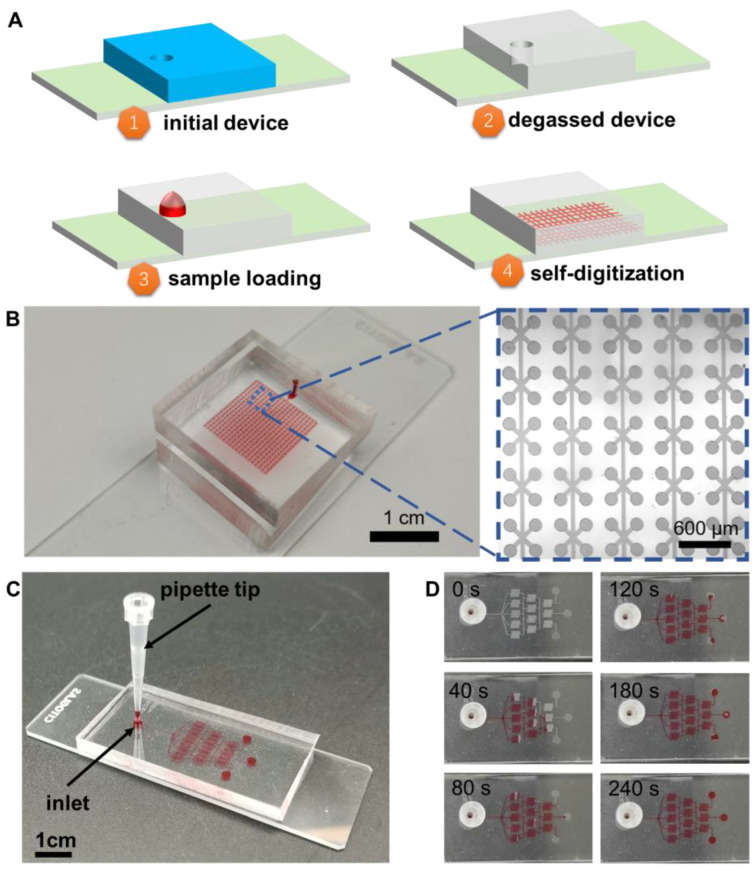
Demonstration of pre-degassed cdPCR microfluidic device. (**A**) Working principle of the pre-degassed cdPCR device. (**B**) Left: a cdPCR microfluidic chip loaded with red dye as an indicator; here, 40 × 40 chambers were fabricated. Right: microscopic view of functional units. (**C**,**D**) Demonstration and time slicing of sample loading and self-digitization process by pre-degassing. Here a pre-degassed PDMS microfluidic chip with a large flow resistance was used to highlight the sample loading process.

Figure 1A illustrates the working principle of the pr-degassed cdPCR microfluidic chip, as used in our recent works [27,28]. There are 40 × 40 digital chambers (150 μm diameter, 30 μm height) within the device, which is fabricated by soft lithography. As shown in Figure 1A, the initial device was prepared by a plasma bonding of a PDMS channel and a glass slider. Then, the whole device was degassed for 30 min at −1 kPa in a vacuum box (Fujiwara PC-3, Taizhou, China). Due to the air permittivity of the PDMS block [29,30], the degassed PDMS chip was compressed in order to provide negative pressure. The appearance and microscopic view of the real device are shown in Figure 1B. Once the 1.5 μL PCR mixture reagent is loaded onto the inlet, it flows automatically due to the negative pressure condition of the cdPCR device. To note, the degassing performance is well related to the degassing time and negative pressure level. In our design, the sample loading and self-digitization could be completed within 2~5 s due to the low flow resistance of the cdPCR device. In other words, the reagent loading and self-digitalization operation could be slowed down if we cut the degassing time, pre-degassed with a smaller negative pressure, or used a PDMS device with a higher flow resistance. Figure 1C shows a pyramid-like PDMS device with a pipette tip. The microchannel here is 50 μm in width and 10 μm in height, and thus it is expected to have a higher flow resistance than the 40 × 40 cdPCR device. Although the device is commonly used as a concentration maker in most cases, here we used this device to better understand the self-digitalization and reagent loading process. To clearly show the reagent loading and self-digitization, we provide a demonstration of the self-digitization process using a concentration gradient generator, as shown in Figure 1C,D. Figure 1B demonstrates the 40 × 40 chamber basic unit for self-digitalization, while Figure 1C,D shows the sample loading process. The demonstration device is patterned with a classical christmas-tree structure microchannel, and it only has one inlet. With three blind outlets, the pre-degassed microfluidic device enables sample loading from the pipette tip. It is important to note that while the reagent could potentially be injected into the microchambers by pushing the pipette with less operation time, doing so may result in the formation of air bubbles. To address this issue, it is still suggested to load the reagent samples following the instructions provided in this work.

### 2.3. Multiplexed Imaging and Data Processing

**Figure 2:**
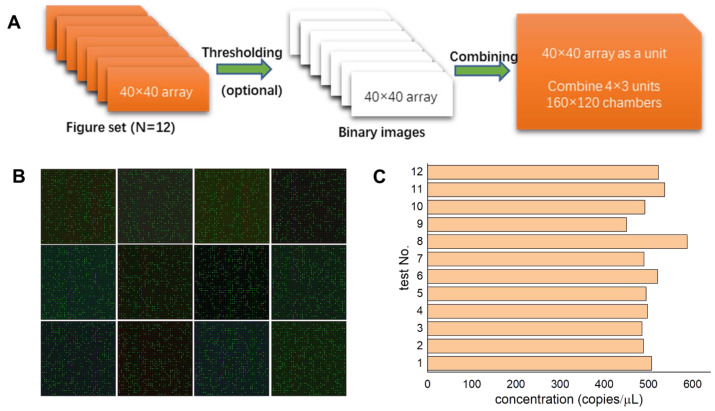
Principle and verification of virtual multiplexing cdPCR tests. (**A**) The conception of virtual multiplexing. (**B**) The fluorescence image sets of DNA templates from cow milk. (**C**) The calculated copy numbers of the DNA template in (**B**) show the coefficient of variations among 12 separate cdPCR tests.

In our previous work, all the reactions took place within 40 × 40 chambers. To improve the detection accuracy, it is suggested to scale up the chamber counts to ~20,000. On the other hand, the total volume of the cdPCR space (including the chambers and dead volumes) is around 2 µL only, which is much smaller than traditional 20 µL PCR reagents. Following this concept, we enlarged the chamber amount by virtual multiplexing. As shown in Figure 2A, we collected fluorescence images from 12 tests using a cdPCR device with the same design. Thus, there would be 40 × 40 × 12 chambers in all. Following this procedure, there would be 160 × 120 digital chambers used for data analysis, even though we did fabricate a real cdPCR device with 160 × 120 chambers. The multiplexing image processing methodology proposed in this work could be defined as a virtual multiplexing cdPCR conception.

As demonstrated in Figure 2B, we carried out 12 independent tests using cow milk DNA samples and obtained these fluorescence images after thermal cycles. Images were captured by a commercial CCD (Charge-coupled Device) camera (Nikon DS-Qi2, Tokyo, Japan) installed on an inverted fluorescence microscope (Nikon Eclipse Ti-S, Tokyo, Japan) and then further analyzed by ImageJ software. Using statistical analysis on the grayscale or fluorescence intensity of microchambers, a threshold value is suggested to distinguish the positive chamber from the negative ones.

For each cdPCR device, the concentration of target DNA templates, $N_{cal}$, was calculated from Poisson distribution and by the following Equation (1) [11],

(1) $$N_{cal}=\frac{\lambda }{v}=\frac{-ln(1-N_{read}/n)}{\pi D^{2}h/4}$$

where *λ* and *v*, respectively, stand for wavelength and chamber volume. $N_{read}$ is the number of positive chambers from the experimental fluorescence images; n is the total number of droplets (here *n* = 1600 for each 40 × 40 cdPCR device); *D* and *h* are the diameter and height of the microchamber.

In this work, the concentration of the target DNA templets using virtual multiplexing images could be calculated as:

(2) $$N_{multiplex}=\frac{-ln(1-\sum_{i=1}^{m}N_{read(i)}/1600\;m)}{\pi D^{2}h/4}$$

where *m* stands for the amount of 40 × 40 chamber cdPCR devices.

## 3. Results and Discussion

### 3.1. Benefits of Virtual Multiplex Imaging

As shown in Figure 2B,C, the number of positive chambers of the 12 fluorescence images in separate tests are 377, 365, 363, 371, 369, 386, 366, 428, 340, 367, 396, and 387, respectively. Based on Equation (1), the calculated concentration of the target cow milk DNA template (expected concentration is 500 copies/μL, measured by an ultraviolet spectrophotometer) is 507, 488, 485, 498, 495, 521, 490, 587, 451, 491, 536, and 522 copies/µL. Thus, the coefficient of variations (CV) of DNA template copy numbers is 6.37%. If we regard these images as a virtual multiplex image series, there would be 4515 positive chambers among the 19,200 chambers; thus, the calculated target DNA concentration would be 506 copies/μL. It can be concluded that the calculated DNA template concentration from virtual multiplexing data is very close to the expected values.

Considering the flow resistance of ultra-large-scale integration of real multiplex cdPCR microfluidic device, the efficiency of the pre-degassed sample loading method is limited due to the poor matching between driven pressure and flow resistance. Therefore, another benefit of virtual multiplexing is that it is not required to redesign or fabricate any 160 × 120 chamber cdPCR microfluidic devices, making the virtual multiplexing easy to be completed using 12 detection units which consist of 40 × 40 microchambers. To note, the number of total chambers could be determined case by case, and it depends on the requirement of the accuracy of applications.

### 3.2. Validation with ddPCR

**Figure 3:**
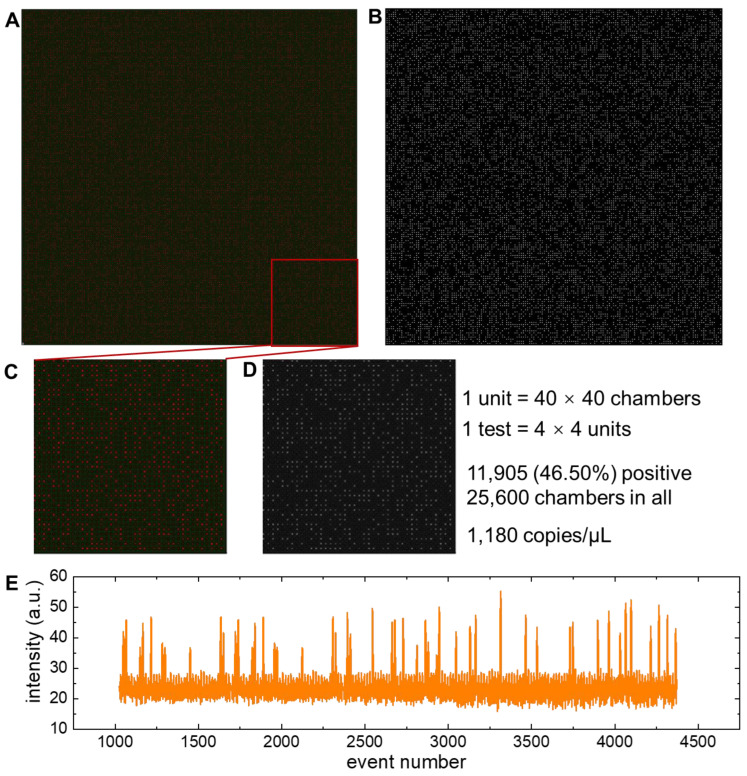
Principle and verification of virtual multiplexing cdPCR tests. (**A**) The fluorescence image from 160 × 160 chambers virtual multiplexing cdPCR microfluidic device and its (**B**) binary image. (**C**) The fluorescence image of a 40 × 40 chamber cdPCR microfluidic chip and its (**D**) binary image as a basic reaction unit. (**E**) The fluorescence intensity as a function of event number in a ddPCR test.

To verify the quantitative analysis ability of virtual multiplexing cdPCR devices, we demonstrated ddPCR tests using commercial equipment. The DNA template used in this work was from camel milk, and the probes were labeled with CY5 dye (650/670 nm). Figure 3A shows a fluorescence image of a virtual multiplexing cdPCR with 160 × 160 digital chambers. With the image stitching, the virtual multiplexing cdPCR image was carried out. Here the image was mosaiced from 16 independent fluorescence images of 40 × 40 chambers. After image binarization and data visualization, the positive or negative chambers could be well distinguished, as shown in Figure 3B.

Correspondingly, Figure 3C,D shows the chambers in a typical 40 × 40 chamber reaction unit. From the binary image in Figure 3D, it can be seen that there were 11,905 positive chambers among 25,600 chambers, and thus the initial concentration of the camel DNA template was calculated as 1180 copies/μL. As a comparison, the same sample was analyzed by the Biorad ddPCR system, and Figure 3E provides a typical curve of real-time fluorescence intensity as a function of time. As a validation, the calculated template concentration was 1193 ± 15 copies/μL.

### 3.3. Applications in Milk Authentication

**Figure 4:**
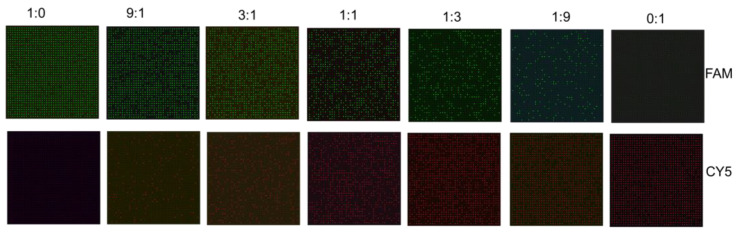
Typical fluorescence images of standard milk samples after amplification.

To demonstrate the performance of milk authentication, Figure 4 shows the fluorescence images of standard milk samples prepared following the ratios in Section 2.1. Among the standard milk samples, from left to right, the volume ratio of cow milk to total volume is 100%, 90%, 75%, 50%, 25%, 10%, and 0%, respectively. The figures presented here are intended solely for demonstration purposes, depicting the 40 × 40 chambers. The label on the top stands for the cow/camel volume ratios. The subfigures in the top line display images from the FAM channel, where the cow milk components could be detected. On the other hand, the subfigures in the bottom line display images from the CY5 channel where the camel milk components could be detected. It can be seen that the numbers of positive chambers in Figure 4 of cow/camel milk are 1491/0, 1290/150, 1089/370, 730/703, 354/1038, 172/1295, and 0/1442, respectively. Subsequently, it could be predicted that the cow milk ratios of these samples are around 100%, 89.58%, 74.64%, 50.94%, 25.43%, 11.72%, and 0%, respectively. The calculated results of the experiments agreed with the predicted ones.

**Figure 5:**
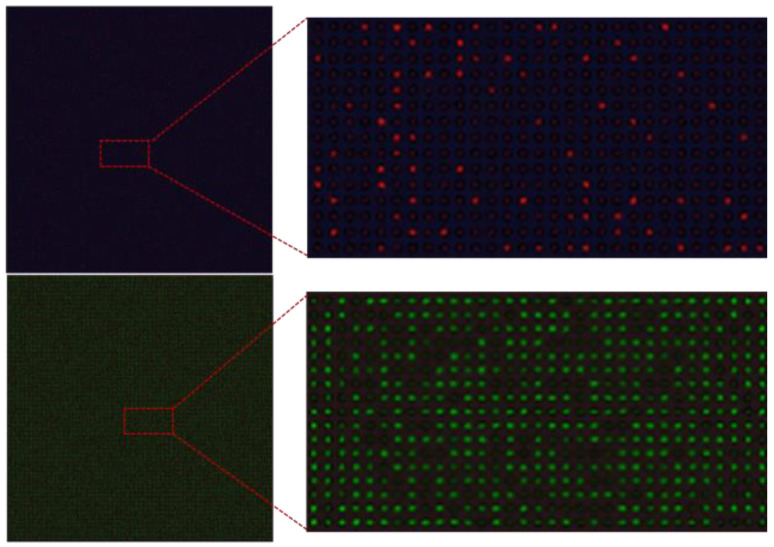
Virtual multiplexing cdPCR applied in cow/camel milk authentication.

During the blind test, pre-mixed cow-camel milk samples were evaluated to predict the mass ratio of camel milk ingredients present in the mixture. The purpose of this test was to assess the ability of the testing method to accurately determine the proportion of camel milk components in the mixed sample without prior knowledge of the actual ratios. The experiments were carried out using the 40 × 40 chamber microfluidic devices to realize a 160 × 160 chamber virtual multiplexing cdPCR test. Taking significant financial profit into consideration, the threshold mass ratio for milk authentication is set at approximately 10%. This means that if the proportion of non-camel milk ingredients in a mixed sample exceeds 10%, it would be considered adulterated or inauthentic. Figure 5 shows that there were 3560 positive chambers in the CY5 channel and 18,371 positive chambers in the FAM channel. The virtual multiplexing blind test indicates that the ratio of the camel milk ingredient is about 10.57~16.23%, which is much lower than 90%, indicating that the mixed milk is not pure camel milk.

## 4. Conclusions

In summary, we presented a virtual multiplexing chamber-based dPCR microfluidic device (i.e., 160 × 160 chambers) with 40 × 40 microchambers for camel/cow milk authentication, which completed the function of self-digitalization, reagent loading, DNA amplification, fluorescence imaging, and image processing. We showed that our virtual cdPCR microfluidic device provided a straightforward way to take ultra-high throughput by using tens of 40 × 40 chamber cdPCR devices. We further showed that our chamber-based virtual multiplexing cdPCR device was able to improve detection accuracy by increasing the number of basic units rather than remaking a cdPCR chip with more microchambers. The benefits of virtual multiplexing conception are to cut down the fabrication cost and improve the performance of pre-degassing and thus the reagent loading and self-digitalization. Moreover, the device is available to be applied in milk authentication or other bio-applications.

## Figures and Tables

**Table 1:** Primer and probe sequences.

Species	Primers/Probes	Sequence (5′→3′)
Cow milk	Primer upstream	TTAGCAGGCAACCTAGCCCA
	Primer downstream	CGAACAGAGGGGTTTGGTATTG
	Probes	FAM-CTTCAGTAGATCTAACCATT-MGB
Camel milk	Primer upstream	CATCCACAGCAGTCCACACC
	Primer downstream	GGTAGAAGATGTAGGTGGAAGGAC
	Probes	CY5-CCAGCCTCTTCACCAGTATCCCTGACA-BHQ1

## Data Availability

Data are available from corresponding authors for reasonable request.
